# Enhancing the Nucleoside Analog Response with Translational Therapeutic Approaches to Overcome Resistance

**DOI:** 10.3390/cells15020130

**Published:** 2026-01-12

**Authors:** Jenna Thibodeau, Kian Hershberger, Sai Samanvitha M. Ramakrishna, Yongwei Su, Lauren Timmer, Bryce Brophy, Katherine Zhang, Holly Edwards, Jeffrey W. Taub, Yubin Ge

**Affiliations:** 1 Cancer Biology Graduate Program, Wayne State University School of Medicine, Detroit, MI 48201, USA; gw4062@wayne.edu (J.T.); cq7751@wayne.edu (K.H.); hz0248@wayne.edu (S.S.M.R.); 2 MD/PhD Program, Wayne State University School of Medicine, Detroit, MI 48201, USA; 3 Department of Oncology, Wayne State University School of Medicine, Detroit, MI 48201, USA; ha8092@wayne.edu (Y.S.); ai0258@wayne.edu (H.E.); 4 Molecular Therapeutics Program, Barbara Ann Karmanos Cancer Institute, Wayne State University School of Medicine, Detroit, MI 48201, USA; 5 Wayne State University School of Medicine, Detroit, MI 48201, USA; hy9268@wayne.edu (L.T.); hu7571@wayne.edu (B.B.); katherine.zhang@wayne.edu (K.Z.); 6 Division of Pediatric Hematology/Oncology, Children’s Hospital of Michigan, Detroit, MI 48201, USA; 7 Department of Pediatrics, Wayne State University School of Medicine, Detroit, MI 48201, USA

**Keywords:** nucleoside analogs, chemotherapy resistance, small molecule inhibitor, transport, deoxycytidine kinase, DNA damage response, BCL-2 proteins, metabolic reprogramming

## Abstract

**Highlights:**

**What are the main findings?**

- The anti-tumor efficacy of nucleoside analogs is dependent on transport, enzymatic activation, nucleotide pools and metabolic responses, which are often aberrant in cancer cells, rendering these chemotherapeutic drugs ineffective.

**What are the implications of the main findings?**

- Small molecule inhibitors are being tested/developed to target or downregulate specific mechanisms responsible for resistance to nucleoside analogs. The combination of small molecule inhibitors with nucleoside analogs has enhanced the efficacy of nucleoside analogs and improved patient outcomes.

**Abstract:**

Nucleoside analogs remain central to the treatment of hematologic malignancies and solid tumors, yet resistance frequently occurs, contributing to relapse and disease-related mortality. Rather than arising from a single mechanism, effective nucleoside analog activity requires successful navigation of multiple biological barriers, including cellular uptake, intracellular activation, nucleotide pool balance, genome surveillance, and mitochondrial stress responses. This review integrates recent advances describing how alterations at each of these levels contribute to resistance to nucleoside analog therapies. We further highlight emerging therapeutic strategies centered on small-molecule inhibitors that exploit these vulnerabilities to enhance the efficacy of nucleoside analogs. Together, this integrative perspective supports the need for development of small molecule inhibitors and design of combination approaches aimed at restoring apoptotic competence and improving the use of nucleoside analog-based therapies for the treatment of cancer.

## 1. Introduction

Nucleoside analogs have been critical components of cancer chemotherapy regimens for decades, due to their potent antimetabolite activities. Agents such as Gemcitabine (GEM) and Fluorouracil (5-FU) have been approved by the United States (U.S.) Food and Drug Administration (FDA) for the treatment of many solid tumors, including pancreatic, breast, lung, bladder, esophageal, stomach, and cervical cancer [1,2,3,4,5]. Other agents, including Cytarabine (Ara-C), Azacitidine (AZA), Decitabine (DAC), Cladribine (CdA), Fludarabine (FA), and Nelarabine (Ara-G), are used in the treatment of many liquid tumors, including acute myeloid leukemia (AML), chronic lymphoblastic leukemia (CLL), hairy-cell leukemia (HCL), T-cell acute lymphoblastic leukemia (T-ALL), and T-cell lymphoblastic lymphoma (T-LBL). Each of these drugs operate by direct incorporation into DNA or RNA to cause cell death and/or targeting specific enzymes responsible for DNA replication or methylation that can trigger cell death (Table 1) [6,7,8,9,10,11]. Despite their broad applications in cancer treatment, this class of drugs can encounter several biological and pharmacologic barriers that can diminish their effectiveness. Mechanistically, these limitations arise from multiple distinct pathways that contribute to either intrinsic or acquired resistance.

**Figure 1:**
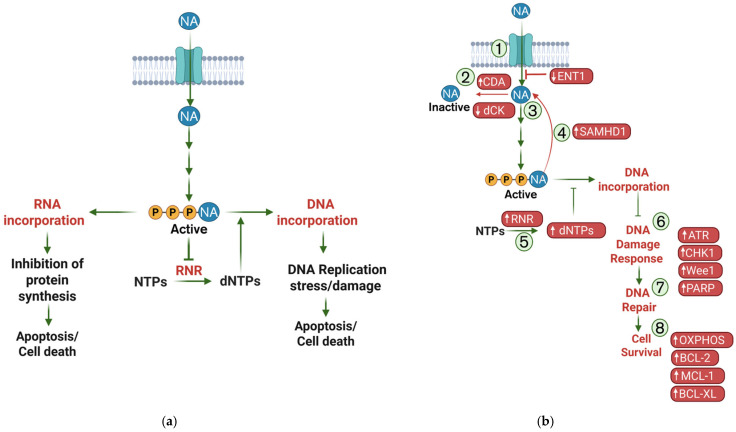
Overview of nucleoside analog mechanisms of action and resistance. (**a**) A generic nucleoside analog (NA) requires multiple coordinated steps to induce cytotoxicity. Following cellular uptake, the analog undergoes phosphorylation catalyzed by kinases, generating triphosphorylated NA (active metabolite) that compete with endogenous dNTPs for incorporation into DNA or RNA. Incorporation of the analog into DNA leads to replication fork stalling, activation of DNA damage response, and, ultimately, cell death. (**b**) Expanded schematic highlighting key molecular regulators of each step and therapeutic targets discussed in this review (highlighted in red). Decrease of ENT1 reduces transport of NA across cell membrane (1). Increased activity of inactivating enzyme, CDA (2), or decreased activity of activating enzyme, dCK (3), reduces NA activation once inside the cell. Hydrolysis of triphosphorylated NA is mediated by SAMHD1 (4), reverting the active drug back to inactivity. Increase in ribonucleotide reductase (RNR) activity increases the dNTP pools, increasing their likelihood of incorporation, thereby decreasing triphosphorylated NA incorporation (5). Increase in the DNA damage response proteins (ATR, CHK1, Wee1, and PARP) (6) allow for cell cycle arrest, DNA repair, DNA replication (7), and cancer cell survival (8). Additionally metabolic reprogramming, such as an increase in OXPHOS and/or upregulation of the anti-apoptotic BCL-2 family members BCL-2, MCL-1, and BCL-XL, enhance cancer cell survival from NA treatment.

Because cancer remains the second leading cause of death in the U.S., continuous efforts are directed toward improving therapeutic responses in both solid tumors and hematologic malignancies [12]. There are two main approaches to identify and target vulnerabilities and eliminate chemoresistant cancer cells. The first approach is to discover small molecule compounds to target dependencies in chemoresistant cancer cells but spare normal cells from cytotoxic effects. Another approach is to develop rationally designed combination therapies to resensitize chemoresistant cancer cells to frontline chemotherapy. This can be achieved by using small molecule inhibitors (SMIs) in combination with chemotherapy to inhibit the identified mechanisms of resistance, allowing for increased cytotoxicity [13]. However, this approach with nucleoside analogs is challenging due to their multi-layer mechanism of action, where each layer provides a different barrier that the drug must overcome to have the desired effect on cancer cells [14]. Most often, these drugs must first be transported into the cells via nucleoside transport membrane proteins. Then, the drugs must be phosphorylated by kinases, including the rate-limiting enzyme deoxycytidine kinase (dCK), and converted into their active form. Once activated, the drug competes with naturally occurring nucleotides for incorporation into DNA or RNA, causing stalled replication, DNA damage, and, ultimately, cell death (Figure 1a). This multifaceted mechanism results in barriers such as reduced drug uptake, a defective activating enzyme, and aberrant metabolism inhibiting the downstream effects, resulting in drug resistance (Figure 1b). This review takes a stepwise approach to summarize the current understanding of nucleoside analog resistance, from cellular uptake through to metabolic reprogramming. We discuss the underlying mechanisms, highlight the key molecular components that drive resistance, and describe how these vulnerabilities are currently being therapeutically targeted.

## 2. Reduced Cellular Uptake in Nucleoside Analog Resistant Cancer Cells

Reduced drug uptake is the first major barrier upon administration of nucleoside analogs. The following section will briefly outline the well-known mechanisms of resistance related to the cellular uptake of nucleoside analogs and provide insight into the current therapeutic advancements to improve drug entry to maintain the cytotoxic levels in cancer cells.

### 2.1. Cellular Uptake Mediated by ENT1

The first barrier in the efficacy of nucleoside analogs revolves around the ability of the drugs to reach their targets. Many of these molecules are charged and hydrophilic, which limits their ability to passively diffuse across the cell membrane to achieve an intracellular cytotoxic concentration. Therefore, they rely on the nucleoside transporters that are present on the cell membrane. Loss or downregulation of nucleoside transporters, particularly the equilibrative nucleoside transporter (ENT1), is a well-established mechanism of resistance to nucleoside analogs and is associated with poor prognosis [15,16]. Mechanisms causing the repression of ENT1 are driven by diverse tumor-associated stresses, including epithelial–mesenchymal transition (EMT), hypoxic environments via HIF family transcription factors, and stress-induced signaling pathways induced by chemotherapy [17,18]. These findings have motivated the development of alternative strategies that either bypass the need for transporter function or target the adaptive pathways that suppress its function.

### 2.2. Strategies to Overcome Transport-Mediated Resistance

One major class of transporter-bypass strategies involves ProTide technology, which was discovered in 2008, inspired by the transport- and activation-related challenges presented by nucleoside analogs. The overall goal of ProTides is to deliver pre-activated monophosphate nucleoside analogs intracellularly through passive diffusion. The first GEM ProTide, NUC-1031, showed improved pharmacokinetics and a favorable safety profile across multiple early-phase trials in pancreatic and ovarian cancers, but was terminated in a phase III clinical trial due to concerns of efficacy [19,20]. However, ProTide designs continue to evolve and represent one of the most promising transporter-independent approaches [21]. NUC-3373, a 5-FU ProTide, was administered to patients with advanced solid tumors in a phase I clinical trial, NCT02723240. NUC-3373 displayed a favorable safety profile with prolonged progression-free survival of cancer patients, including those who previously relapsed after 5-FU treatment [22]. In 2025, NUC-3373 progressed to a phase II clinical trial in combination with other agents commonly used to treat colorectal cancer; however, this trial was terminated due to the unlikeliness of achieving the clinical goal. However, NUC-3373 still holds promise to improve the treatment of solid tumors, but more research is needed.

ProTide technology is also being utilized to deliver small molecule inhibitors, along with the nucleoside analog, to improve activity. The ProTide NUC-1031 was conjugated to the CD13 inhibitor, Bestatin, to target CD13 (known to increase upon exposure to GEM and 5-FU and cause resistance) and improve the efficacy of NUC-1031 [23]. Further advancements are now being made by conjugating ProTides to antibodies to improve the delivery of phosphorylated drugs that remain promising to expand to nucleoside analogs [24]. Many pharmaceutical companies are developing novel ProTides for anti-cancer applications centered around nucleoside analogs [25].

In parallel, alternative nucleoside analog delivery systems are being developed. Nucleoside analog-incorporated aptamers are small single-stranded oligonucleotides that have shown a high affinity for target binding [26]. Previously, few aptamers have shown preliminary success against pancreatic and colon cancer models, yet none have been approved for clinical use [27,28,29]. More recently, nanoparticle-based formulations represent another delivery system that can encapsulate nucleoside analogs and bypass transporter dependence. Nanoparticles are small molecules, ranging from 1 to 100 nanometers, that can be synthesized to encapsulate therapeutic agents to improve drug stability and targeted delivery and reduce exposure to surrounding healthy tissues [30]. Nanoparticles can be engineered from various materials, such as lipids, polymers, metals or inorganic materials, depending on the specific application [31]. The flexible framework provides advantages to overcome resistance mechanisms such as drug uptake and tumor penetration, making them an attractive avenue of research in cancer. Like ProTides, nanoparticles have recently been developed to co-deliver SMIs to induce additional cytotoxicity to cancer cells in combination with chemotherapy to enhance drug accumulation and suppress additional resistance mechanisms (Table 2).

A complementary approach is to stimulate ENT1 activity through the activation of signaling pathways. Breast cancer cells treated with a low concentration of protein kinase C (PKC) activator phorbol 12-myristate 13-acetate (PMA) showed an increase of ENT1-mediated uptake of [^3^H]2-chloroadenosine as a proof of concept that PKC regulates ENT1 activity [38]. A CML murine model also showed that activation of the JNK pathway negatively regulates ENT1 expression and activity, therefore identifying another targetable mechanism to potentially increase ENT1 activity [39].

Collectively, these emerging strategies highlight a rapidly evolving therapeutic landscape in which alternative drug delivery platforms bypass, restore, or exploit transporter deficiencies and additionally target other resistance. These developments lay a mechanistically grounded framework for overcoming nucleoside analog resistance across multiple types of cancers.

## 3. Enzymatic Determinants of Nucleoside Analog Efficacy

**Figure 2:**
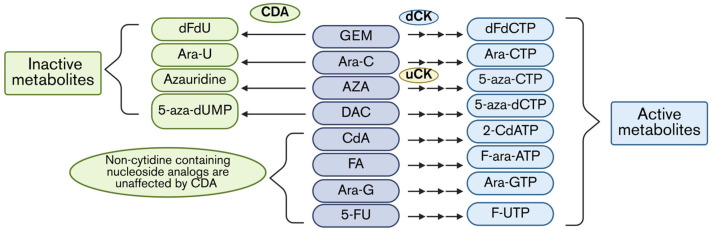
Inactive and active metabolites of nucleoside analog prodrugs via CDA and dCK/uCK activity. Prodrugs are aligned in the middle column, in which dCK or uCK catalyze the rate-limiting step in the activation of each drug, resulting in a triphosphate NA represented in the rightmost column. CDA can deaminate the prodrugs that contain cytidine into an inactive metabolite, shown in the leftmost column.

After uptake, the nucleoside analogs then must undergo phosphorylation mediated by kinases to become active metabolites. dCK catalyzes the rate-limiting phosphorylation of the prodrugs GEM, Ara-C, CdA, DAC, and FA to become monophosphate metabolites, while uridine ctyidine kinase (uCK) catalyzes the rate-limiting phosphorylation of the prodrug AZA to become monophosphate-AZA. These monophosphate nucleoside analogs are further phosphorylated to become triphosphate forms, active metabolites, which are incorporated into DNA or RNA, or inhibit ribonucleotide reductase (RNR) (Figure 1). Thus, defects in dCK-mediated activation represent a second major barrier to effective therapy, with nucleoside analogs [40]. In parallel, cytidine deaminase (CDA) has been shown to be heavily implicated in nucleoside analog resistance, as it can rapidly deaminate cytidine-based prodrugs prior to activation, causing resistance to nucleoside analogs (Figure 2) [40,41]. Therefore, the following sections will discuss the role of enzymes, dCK, and CDA in resistance and advancements in SMI development to improve drug activation inside cancer cells.

### 3.1. Enzymatic Activation—Defects in dCK Cause Resistance

**Figure 3:**
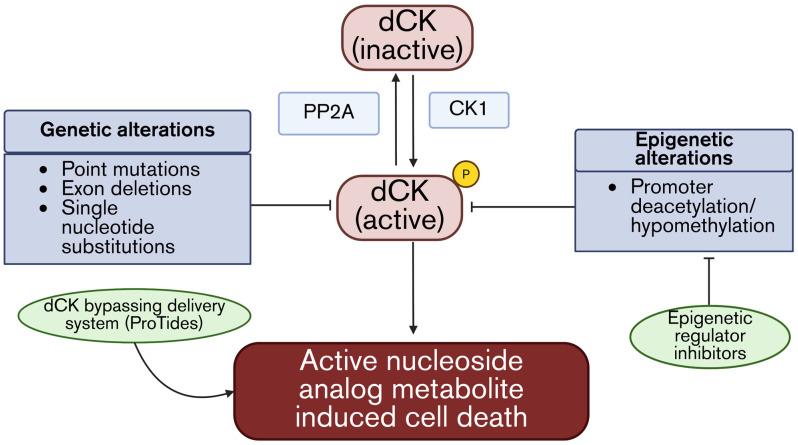
Summary of dCK-mediated resistance mechanisms and overcoming strategies. dCK requires phosphorylation via Casein kinase 1 (CK1) for activation; however, mutations in PP2A remove the phosphate, rendering dCK inactivation. Additionally, dCK activity is controlled by genetic and epigenetic alterations that can be overcome by alternative drug delivery systems or epigenetic regulators to preserve dCK activity and promote active nucleotide analog-induced cell death.

Under the pressure of nucleoside analog exposure, malignant cells have been shown to acquire different types of mutations in the *DCK* gene, including point mutations, frameshift mutations, exon deletions, and splice site alterations that impair the stability or catalytic functions of the dCK enzyme [42]. A recent study in AML reported newly acquired *DCK* mutations after Ara-C exposure [43]. In a separate study of murine AML cell lines, Ara-C resistance was associated with either a large deletion in the splice acceptor of exon 7, leading to alternative splicing, or a frameshift mutation in exon 4 that resulted in a truncated protein. Both cases resulted in nonfunctional dCK proteins that correlated with Ara-C resistance [44]. Unfortunately, there are no drugs with the ability to correct genetic mutations for dCK; therefore, in instances where dCK is genetically mutated, the ProTide or nanoparticle approach is required to bypass this activation step completely (Figure 3).

Post-translational regulation adds yet another layer of complexity. dCK requires phosphorylation at Ser74 by Casein kinase 1 (CK1) for activation. The Ser/Thr phosphatase PP2A removes the phosphorylation at this site, leading to the inactivation of dCK. In up to 40% of uterine serous carcinomas, a mutation in the *PP2A* gene results in a stable variant that confers resistance to both adenosine and cytosine analogs by promoting the dephosphorylation and inactivation of dCK (Figure 3) [45].

However, other studies have demonstrated epigenetic causes for the downregulation of dCK, including deacetylation and hypermethylation of the *DCK* promoter that suppress transcription in resistant AML cell lines [46,47]. Identifying these epigenetic mechanisms is clinically relevant, as they may be exploited for unique therapeutic opportunities. For instance, the FDA-approved SMI, Vorinostat, was found to resensitize the above-mentioned leukemia cells to Ara-G by preventing the deacetylation of H3 and H4 and allowing for the transcription of the *DCK* gene (Figure 3) [48].

Collectively, these alterations converge on reduced dCK activity, limiting the formation of active nucleoside analog metabolites. However, there are instances in which some of the drugs are rapidly inactivated after transport, not allowing for activation to take place.

### 3.2. Enzymatic Inactivation—CDA Activity Causes Resistance

CDA is involved in the pyrimidine salvage pathway, which is essential for the synthesis of DNA and RNA in normal cells. CDA catalyzes the deamination (removal of an amino group) of cytidine or deoxycytidine to uridine or deoxyurdine, respectively. However, like dCK, this function extends to nucleoside analogs, through which they can be deaminated and not given the chance to be activated by dCK or uCK (Figure 2). CDA plays an important role in causing resistance to the cytidine-based analogs: specifically, GEM, Ara-C, DAC, and AZA [49,50,51,52]. Therefore, developing SMIs against CDA represents a promising approach to overcome CDA-mediated resistance to nucleoside analogs.

### 3.3. Advancement in CDA Small Molecule Inhibitors

CDA has been shown to be expressed in intestines, liver, and bone marrow, which can result in rapid deamination of nucleoside analogs [52]. An earlier virtual screening revealed zebularine to be the best candidate against CDA activity [53]. Recently, researchers have tested zebularine in combination with GEM, which resulted in synergy in pancreatic cancer cells, revealing it to be a promising approach in pancreatic cancer treatment in vitro [54].

In 2020, the FDA approved the combination of DAC plus cedazuridine, a CDA small molecule inhibitor, for adult patients with myelodysplastic syndrome or chronic myelomonocytic leukemia (CMML) [55]. This successful combination has sparked further development of CDA inhibitors. Tetrahydrouridine (Thu) is an investigational small molecule inhibitor that is a well-known competitive inhibitor of CDA. It was shown to dramatically increase the DAC concentration in plasma in vivo [56]. There is an open phase I trial (NCT07006025) actively recruiting patients with relapsed or refractory myelodysplastic syndrome to assess toxicity profiles and improvement of health status in response to this combination therapy.

In addition to combination therapy, an alternative approach being explored is the modification of nucleoside analog molecules to resist the deamination via CDA. It was recently discovered that sialylation (addition of salic acid to molecules to improve stability) of DAC or AZA improved stability and activity in both solid and liquid tumors. This discovery led to the use of the small molecule OR21—an oligonucleotide consisting of DAC and 5′-O-trisilylate, which is protected from CDA inactivation and exerts similar anti-cancer activity to DAC, but with less toxicity [57].

The recent development of a real-time fluoresce-based deamination assay which details the exact measurement of CDA kinetics in the presence or absence of inhibitors resulted in a powerful screening technique, demonstrating the relevance and potential that CDA inhibition has for cancer treatment advancements [58]. Overall, CDA is implicated in resistance to nucleoside analogs, and many preclinical and clinical studies are currently investigating ways to inhibit CDA or make the nucleoside analogs resistant to CDA, possessing clinical promise in overcoming resistance to these antitumor agents.

## 4. Altered Nucleotide Metabolism and dNTP Pool Rewiring

Beyond defects in cellular uptake and dCK-mediated activation, cancer cells frequently exploit the downstream nucleotide metabolism to evade nucleoside analog cytotoxicity. Once phosphorylated to their active forms, nucleoside analogs enter the tightly regulated network that is responsible for maintaining balanced deoxynucleotide triphosphate (dNTP) pools, which are essential for DNA replication, repair, and mitochondrial genome maintenance. Here, the triphosphate NAs compete with dNTPs for incorporation into DNA to cause cell cycle arrest and apoptosis. RNR is the rate-limiting enzyme in the biosynthesis of dNTPs, while sterile alpha motif histidine containing protein 1 (SAMDH1) maintains the homeostatic levels of dNTPs. Aberrant function of these crucial enzymes can cause an altered nucleotide metabolism and resistance to nucleoside analogs. Therefore, in this section, we discuss the mechanisms of nucleotide metabolism-related resistance and overcoming strategies.

### 4.1. RNR

RNR is the central control point of de novo dNTP biosynthesis. It converts ribonucleoside diphosphates to deoxyribonucleoside diphosphates and is tightly regulated by allosteric mechanisms and the cell cycle. RNR has two homodimeric subunits: the larger RRM1 regulatory subunit and the smaller RRM2 catalytic subunit [59]. While RRM1 subunit levels are maintained throughout the cell cycle, the RRM2 subunit is regulated so that its expression is increased during the S phase [59,60]. As a result, the RNR activity is highest during the S phase when DNA synthesis is taking place, and the cell requires high levels of dNTPs. Further regulation of RNR via mammalian target of rapamycin (mTOR) has been observed. mTOR is a protein kinase composed of two complexes: mTORC1 and mTORC2. It has been shown that mTORC2 regulates RNR activity through its phosphorylation of the RRM1 subunit, which enhances the interaction between the two RNR subunits [61]. This enhanced interaction is important because RNR activity is dependent on an intact complex of both subunits.

In cancer, RNR is frequently upregulated or hyperactivated. Overexpression of RRM1 and RRM2 correlates with poor outcomes in several solid tumors, including pancreatic and colorectal cancers [62,63]. Because GEM not only incorporates into DNA but also inhibits RNR and depletes dNTP pools, increased RNR expression or mTORC2-driven stabilization of the RNR complex can allow cancer cells to maintain dNTP synthesis despite GEM exposure, promoting resistance [61,63].

### 4.2. SAMHD1

SAMHD1 counteracts RNR by reducing intracellular dNTP pools through the hydrolysis of dNTPs into deoxynucleosides and inorganic triphosphate. This activity is critical for maintaining balanced dNTP levels outside of S phase and proper cell-cycle progression. Loss or inhibition of SAMHD1 results in expanded and imbalanced dNTP pools, which disrupt normal DNA metabolism [60].

Clinically, low SAMHD1 expression is associated with longer overall survival in several cancers, which is consistent with its role in limiting nucleoside analog efficacy [64,65]. Importantly, SAMHD1 selectively inactivates certain nucleoside analogs by hydrolyzing their triphosphate forms. This selectivity depends on the chemical structure of the drug, particularly modifications to the sugar moiety. As a result, triphosphate-Ara-C and -FA are hydrolyzed by SAMHD1, whereas -GEM is not [64,66].

The coordinated regulation of dNTP synthesis and degradation critically determines the intracellular active metabolites and efficacy of nucleoside analogs. RNR and SAMHD1 play opposing roles in this process, with RNR driving dNTP biosynthesis and SAMHD1 limiting dNTP and analog triphosphate availability through hydrolysis. Dysregulation of this axis therefore provides multiple mechanisms through which cancer cells evade nucleoside analog cytotoxicity, while simultaneously revealing therapeutic vulnerabilities that can be exploited.

Pharmacologic modulation of RNR has uncovered a functional interaction between RNR and SAMHD1 that is particularly relevant in the context of Ara-C resistance. Treatment of AML cells with RNR inhibitors does not simply reduce the total dNTP abundance but instead alters dNTP pool ratios. Under basal conditions, dCTP represents the smallest dNTP pool; following RNR inhibition, dCTP levels increase relative to dATP, resulting in an inversion of the normal dATP/dCTP ratio [67]. This altered nucleotide balance favors the occupancy of allosteric site 2 of SAMHD1 by dCTP: a configuration that fails to fully activate the enzyme [68]. Consequently, SAMHD1-mediated hydrolysis of Ara-CTP is attenuated, allowing for greater intracellular accumulation of the active metabolite and enhanced DNA incorporation. Through this mechanism, RNR inhibition indirectly suppresses SAMHD1 activity, providing an explanation for the ability of RNR inhibitors to resensitize AML cells to Ara-C.

### 4.3. Therapeutic Targeting of the RNR–SAMHD1 Axis to Overcome Nucleoside Analog Resistance

Hydroxyurea (HU) is among the few FDA-approved RNR SMIs [69]. HU combination with Ara-C has shown promising results in a phase I clinical trial. All nine patients with newly diagnosed AML treated with HU and Ara-C achieved complete remission, with no unexpected toxicities observed, and pharmacokinetic analysis revealed increased median Ara-CTP levels [70]. Ex vivo analyses further confirmed enhanced Ara-C sensitization following HU treatment, supporting the translational relevance of this strategy.

Another SMI, BBI-825, has recently undergone an open-label, multicenter, first-in-human, dose-escalation and dose-expansion Phase 1/2 study (NCT06299761) examining the safety profile of BBI-825 as a monotherapy and in combination with selected targeted therapies. This trial was completed in June of 2025 and the results have not been published yet. However, this recent trial indicates that RNR inhibition is still being studied to improve cancer therapeutics.

An alternative targeting strategy involves targeting upstream regulators of RNR activity. As mentioned, mTORC2 enhances the interaction between the RRM1 and RRM2 subunits of RNR, stabilizing the active enzyme complex and sustaining dNTP biosynthesis under genotoxic stress. This stabilization contributes to GEM resistance by enabling continued nucleotide production despite its RNR inhibitory function. Dual inhibition of mTORC1 and mTORC2 using PP242 disrupted the RNR subunit interactions in vivo and significantly improved GEM efficacy without added toxicity, establishing mTORC signaling as a therapeutically relevant regulator of nucleotide metabolism [61].

Additionally, the direct targeting of SAMHD1 represents another complementary approach. Reduced SAMHD1 expression has been consistently associated with increased sensitivity to Ara-C and improved clinical outcomes in AML [64,65]. One experimental strategy to achieve this effect involves Vpx-containing virus-like particles (Vpx-VLPs), which induce proteasomal degradation of SAMHD1. In ex vivo AML models, combined treatment with Ara-C and Vpx-VLPs resulted in significantly increased apoptosis compared to Ara-C treatment alone [64]. Although Vpx-VLP delivery is not yet clinically feasible, ongoing efforts to develop simplified VLP-based delivery systems underscore the therapeutic potential of pharmacologically reducing SAMHD1 [71].

Collectively, studies investigating overcoming resistance at the RNR–SAMHD1 axis involve the inhibition of dNTP synthesis, disruption of SAMHD1 activation or stability, and modulation of upstream signaling pathways. Rational combination strategies that pair nucleoside analogs with agents targeting RNR, mTORC2, or SAMHD1 may therefore represent an effective means of overcoming nucleoside analog resistance in cancer.

## 5. DNA Damage Response

**Figure 4:**
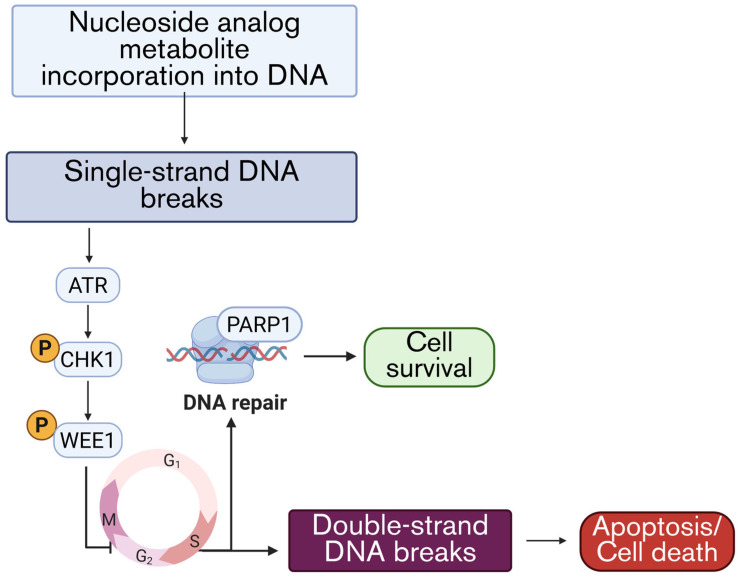
Key components of nucleoside analog-induced DNA damage response. Incorporation of nucleoside analogs into DNA induces single-strand breaks after one round of the cell cycle. ATR senses the breakage and activates CHK1 and WEE1, leading to paused replication in the G2M transition phase. This stalled replication triggers DNA repair mechanisms mediated by PARP, resulting in cell survival; continuation of the cell cycle leads to double-strand breaks and, ultimately, cell death.

Once a nucleoside analog successfully outcompetes endogenous dNTPs for incorporation into DNA, it induces stalled replication forks, mispaired bases, chain termination, or incorporation-induced structural abnormalities. As a result, cells activate the DNA damage response (DDR): a coordinated signaling network that detects DNA lesions, stabilizes replication forks, arrests cell-cycle progression, and initiates DNA repair to promote survival [72]. In chemoresistance, the DDR is often overactive, allowing cancer cells to repair the DNA damage caused by chemotherapy and continue proliferation. This section will discuss the key components of the DDR and efforts to develop inhibitory strategies for these critical players (Figure 4).

### 5.1. Key Components of the DDR

The first tier of the DDR consists of sensor kinases that detect distinct forms of DNA damage or replication stress. Nucleoside analogs often work through the induction of replication stress, which is categorized by stalled or slowed replication forks and the accumulation of single-stranded DNA. This stress is primarily sensed by the ATR kinase, which is activated in response to single-stranded DNA coated by replication protein A (RPA) at stalled forks [73,74]. ATR signaling subsequently activates downstream effector kinases such as checkpoint kinase 1 (CHK1), thereby triggering S and G2/M checkpoints, stabilizing replication forks, and limiting the propagation of damaged DNA. Activated CHK1 activates the WEE1 kinase, which plays a critical role in enforcing the G2/M checkpoint by inhibiting cyclin-dependent kinase 1 (CDK1) activity, preventing cells from entering mitosis with damaged or incompletely replicated DNA [75]. This checkpoint is particularly important in cells experiencing replication stress, such as those treated with nucleoside analogs. Activation of WEE1 therefore provides an additional survival advantage by allowing time for DNA repair and fork recovery, rather than cell death [76]. Through this pathway, cancer cells can tolerate high levels of nucleoside analog-induced DNA lesions, contributing to therapeutic resistance [77].

At sites of replication stress and checkpoint activation, cells must determine whether stalled replication forks can be stabilized and repaired or whether the damage will progress toward cytotoxic outcomes. One adaptive response involves the engagement of downstream DNA repair pathways, including base excision repair (BER) and single-strand break repair. Poly(ADP-ribose) polymerase 1 (PARP1) is a key mediator of these processes, functioning to coordinate repair at replication-associated lesions and stalled forks. Through this activity, PARP1 facilitates the resolution of single-strand DNA damage and limits the conversion of replication stress into lethal double-strand breaks [78].

Collectively, the DDR network, centered on ATR/CHK1/WEE1 signaling, and PARP-dependent DNA repair, represent adaptive survival mechanisms that limit nucleoside analog efficacy. Cancer cells that rely heavily on these pathways to tolerate replication stress are therefore particularly vulnerable to DDR inhibitor-based combination strategies [79]. Targeting ATR, CHK1, WEE1, or PARP has emerged as a rational approach to disable DNA damage tolerance, force checkpoint bypass, and drive lethal genomic instability in nucleoside analog-treated cells, providing a strong mechanistic rationale for employing SMIs against the key DDR component to improve the antitumor activities of nucleoside analogs.

### 5.2. Targeting Key DDR Components to Overcome Resistance to Nucleoside Analogs

Pharmacologic inhibition of ATR disrupts the earliest stage of replication stress sensing, preventing the stabilization of stalled replication forks and promoting replication fork collapse. In nucleoside analog-treated cancer cells, loss of ATR activity results in the accumulation of double-strand DNA breaks and heightened genomic instability. Supporting this concept, a virtual screening effort identified multiple FDA-approved compounds that were capable of interfering with ATR–CHK1 signaling, including quinacrine and thimerosal [80]. In the same study, quinacrine was shown to enhance 5-FU efficacy in mouse models [80]. In parallel, several selective ATR inhibitors have demonstrated preclinical synergy with nucleoside analogs; for example, AZD6738 enhanced GEM to induce marked tumor regression in vitro and in vivo [81].

Clinical translation of ATR inhibition has further strengthened this rationale. Berzosertib, a first-in-class ATR inhibitor, was evaluated in a phase Ib clinical trial in combination with GEM in patients with non-small-cell lung cancer (NSCLC), demonstrating a favorable safety profile alongside encouraging clinical activity. Berzosertib was discontinued before proceeding to the phase II clinical trial; however, the development sparked more interest in SMIs against ATR to treat cancer (Table 3) [82]. These findings highlight ATR inhibition as a promising strategy to disable replication stress tolerance in nucleoside analog-treated tumors.

Given that ATR directly activates CHK1, targeting CHK1 itself provides an alternative means of collapsing checkpoint control. CHK1 inhibition abolishes S-phase and G2/M checkpoints, driving cells through the cell cycle with unresolved DNA damage. Prexasertib, a first-in-class dual CHK1/CHK2 inhibitor, demonstrated clinical activity and tolerability in a subset of ovarian cancer patients as a monotherapy, establishing proof-of-principle for checkpoint abrogation strategies [83]. Although many early CHK1 inhibitors encountered limitations due to off-target toxicity, renewed drug development efforts have focused on increasing selectivity and potency with several clinical trials (Table 3).

CHK1 signaling also feeds into the activation of WEE1, positioning WEE1 inhibition as another effective strategy to abrogate damage tolerance. Pharmacologic WEE1 inhibition forces premature mitotic entry despite incomplete DNA replication, triggering mitotic catastrophe under the conditions of nucleoside analog-induced replication stress. The WEE1 inhibitor adavosertib (AZD1775) has shown strong synergy with Ara-C in AML models, while similar combinatorial effects have been observed in solid tumors using it in combination with GEM across pancreatic, lung, and ovarian cancers [84,85,86,87,88] (Table 3).

Importantly, WEE1 stalls the cell cycle, creating a permissive environment for DNA repair, rather than directly resolving replication-associated lesions. At these stabilized replication forks, PARP1 coordinates repair processes that facilitate lesion resolution and limit fork collapse. In this context, PARP inhibition compromises the repair of nucleoside analog-induced DNA damage, allowing replication-associated lesions to persist, propagate, and ultimately drive cell death. Unlike many checkpoint inhibitors that remain largely in preclinical or early clinical development, PARP inhibitors Niraparib, Olaparib, Rucaparib, and Talazoparib have been FDA-approved since 2014 and are widely used in the maintenance therapies of solid tumors [89]. PARP inhibitors, Olaparib and Talazoparib, have been shown to synergize with GEM by allowing single-strand DNA breaks to progress to double-strand DNA breaks and ultimately cell death [90]. Increasingly, their application is being extended to hematologic malignancies, with multiple studies demonstrating enhanced leukemia cell death when PARP inhibitors are combined with Ara-C [91].

While the DDR is classically viewed as a nuclear signaling network, emerging evidence demonstrates that DDR signaling also extends beyond the nucleus to regulate mitochondrial fate decisions. Notably, alterations in ATR signaling have been shown to promote a mitochondria-localized, anti-apoptotic form of ATR that enforces apoptotic dormancy despite persistent DNA damage [92]. These findings highlight a critical conceptual shift: resistance to nucleoside analogs is not solely determined by the DNA repair capacity but also by mitochondrial rewiring that uncouples the replication stress from cell death [93].

**Table 3:** Clinical trials investigating SMIs against DDR components.

SMI	Target	Trial Details: ID; Phase; Cancer Type; Drug Combination (If Any)	Study Duration	Status/Outcomes
Berzosertib	ATR	NCT04216316 Phase 1b; NSCLC; combination with carboplatin, gemcitabine, and pembrolizumab	2021–2024	Completed; combination was tolerable with clinical activity [82]
ATG-018	ATR	NCT05338346 Phase I open label; solid and hematological malignancies; monotherapy	2022–2024	Terminated due to lack of activity
Camonsertib (RP-3500)	ATR	NCT04497116 Phase 1/2a multi-center, open-label, dose-escalation, and expansion study; advanced solid tumor; combination with PARP inhibitor or GEM	2020–2025	Completed; clinical benefit observed with increased benefit in ovarian cancer [94].
Elimusertib	ATR	NCT04514497 Phase I; NSCLC; combination with chemotherapy	2021–ongoing	Ongoing
Prexasertib	CHK1	NCT02873975 Phase II open label; advanced solid tumors with replicative stress, homologous recombination deficiency, or CCNE1 amplification; monotherapy	2016–2021	Completed; results are expected soon
GDC-0575	CHK1	NCT01564251 Phase I open label dose escalation study; refractory solid tumors or lymphoma; combination with GEM	2012–2018	Completed; observed antitumor activity with manageable hematological toxicities [95].
APR-1051	WEE1	NCT06260514 Phase 1, open-label, multicenter, first-in-human study; advanced solid tumors; monotherapy	2024–ongoing	Recruitment phase
Adavosertib (AZD-1775)	WEE1	NCT02037230 Phase I dose escalation study; pancreatic cancer; combination with gemcitabine (+radiation)	2014–2018	Completed; overall survival is substantially higher than gemcitabine with radiation therapy [96].

## 6. Mitochondrial and Metabolic Reprogramming

Metabolic processes such as nucleotide, amino acid, and lipid metabolism are also required for sustaining malignant proliferation [97]. These biological processes are fueled by mitochondria as the central hubs of bioenergetics, redox balance, and biosynthesis in most eukaryotic cells. Through oxidative phosphorylation (OXPHOS), mitochondria generate ATP and support the metabolic pathways required for cellular energy needs. It was shown that the residual leukemic cells after chemotherapy display high OXPHOS, as well as nucleotide and glutathione synthesis [98]. Mechanistically, transcriptional programs that enhance OXPHOS are implicated in generating a metabolic state that promotes Ara-C resistance [99].

### 6.1. Mitochondrial Metabolism in Resistance

Mitochondrial metabolism plays a crucial role in the response to nucleoside analogs in both hematologic malignancies and solid tumors. As mentioned, inactivation of dCK is a major contributor to GEM and Ara-C resistance. Further, pancreatic cancer cells with dCK inactivation exhibit heightened sensitivity to mitochondrial metabolic disruption [100]. In AML, Ara-C-resistant cell populations frequently display an OXPHOS-high phenotype and cells with hyperactive OXPHOS are selectively vulnerable to OXPHOS and electron transport chain complex I inhibition [101,102,103]. In solid tumors such as pancreatic ductal adenocarcinoma (PDAC), metabolic reprogramming toward enhanced mitochondrial activity likewise drives GEM resistance [104]. In relapsed AML models, Ara-C-resistant cells shift toward mitochondrial metabolism and robust ROS detoxification, further emphasizing mitochondrial plasticity as a resistance mechanism [105]. These findings highlight that mitochondrial metabolism, dynamics, and signaling-mediated metabolic reprogramming are central determinants of resistance to nucleoside analogs in both hematologic malignancies and solid tumors, suggesting that targeting mitochondrial pathways may improve therapeutic efficacy with this class of agents.

### 6.2. Mitochondrial Targeting Strategies

Given the important role of mitochondrial metabolism in resistance to nucleoside analogs, its targeting represents a promising strategy to overcome nucleoside analog resistance. After treatment with chemotherapeutics such as Ara-C, drug-persisting leukemic cells exhibit an increased dependence on mitochondrial function [106]. AML cells depend on α-ketoglutarate to sustain OXPHOS; thus, inhibition of α-ketoglutarate dehydrogenase (OGDH), the rate-limiting enzyme in the tricarboxylic acid (TCA) cycle, with the imipridone ONC213, induces mitochondrial stress and suppresses OXPHOS, leading to antileukemic efficacy both in vitro and in vivo [107]. Other TCA cycle enzymes also represent potential therapeutic targets. GEM exposure elevates reactive oxygen species and enhances TCA cycle flux in pancreatic cancer cells, partly through upregulation of wild-type isocitrate dehydrogenase 1 (IDH1); IDH1 inhibition potentiates cytotoxic responses to GEM [108]. Additionally, in OXPHOS-high pancreatic cancer cell lines, complex inhibitors of the electron transport chain (ETC), such as phenformin, overcome GEM resistance [109], demonstrating that this vulnerability may apply to several cancer types.

Beyond metabolism, mitochondrial genetic programs also influence therapeutic outcomes. Mammalian mitochondrial DNA (mtDNA) encodes 13 polypeptides that form essential components of ETC complexes, including complexes I, III, IV, and ATP synthase [110]. Mitochondrial transcription factor A (TFAM) is essential for mtDNA replication, transcription, and maintenance, thereby supporting proper mitochondrial biogenesis and function. TFAM inhibition has been shown to enhance GEM cytotoxicity and suppress the growth of GEM-resistant pancreatic cancer cells. Mechanistic studies indicate that knockdown of TFAM induces profound mitochondrial dysfunction and energy crisis, followed by increased oxidative stress [111]. In addition, targeting mitochondrial RNAs (mtRNAs) is also a potential therapeutic strategy. Disruption of mitochondrial function via the downregulation of mature mtRNA significantly enhances hypomethylating agents (HMA; AZA and DAC)-induced cell death, underscoring the role of mtRNA-driven mitochondrial activity in HMA resistance [112].

While therapeutic targeting of mitochondrial metabolism and biogenesis can erode the bioenergetic fitness of nucleoside analog-resistant cells, mitochondrial dysfunction alone is often insufficient to ensure cell death. Instead, survival under sustained replication and metabolic stress ultimately depends on active suppression of mitochondrial apoptosis, a process governed by the BCL-2 family proteins.

### 6.3. Anti-Apoptotic BCL-2 Family Proteins-Mediated Resistance

Mitochondrial and metabolic reprogramming ultimately converges on the regulation of mitochondrial apoptosis, a process governed by the BCL-2 family proteins. These proteins function to integrate signals derived from DNA damage, replication stress, metabolic imbalance, and microenvironmental cues to determine cell fate [113,114]. The BCL-2 family is broadly divided into anti-apoptotic members (including BCL-2, BCL-XL, and MCL-1), pro-apoptotic effectors (BAX and BAK), and BH3-only proteins (such as BID, BIM, PUMA, and NOXA), which collectively regulate mitochondrial outer membrane permeabilization (MOMP) and commitment to apoptosis [115].

**Figure 5:**
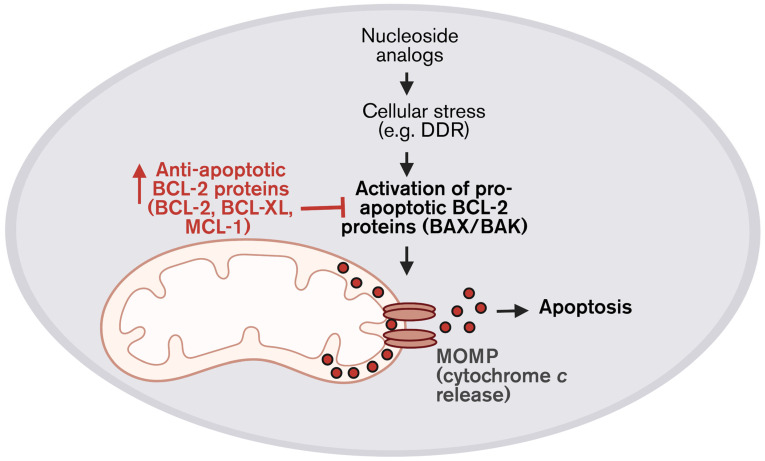
BCL-2 family proteins regulate cellular response to nucleoside analogs. Cellular stress, including DDR, induced by nucleoside analogs activate pro-apoptotic proteins, BAX/BAK, leading to mitochondrial outer membrane permeabilization and cytochrome *c* release (red dots), and, ultimately, apoptosis. Resistant cancer cells can often evade apoptosis by increasing their dependence on anti-apoptotic BCL-2 family proteins (BCL-2, BCL-XL, and MCL-1) that inhibit the activation of BAX/BAK, thereby promoting survival under nucleoside analog stress.

Under nucleoside analog treatment, cancer cells experience sustained replication stress and metabolic perturbation that would ordinarily prime cells for apoptosis. However, resistant cells frequently adapt by increasing their dependence on anti-apoptotic BCL-2 family members, thereby decreasing pro-apoptotic signaling and thus promoting survival despite persistent DNA damage and mitochondrial dysfunction. This implicates BCL-2 family proteins as critical downstream effectors of chemoresistance, rather than upstream drivers (Figure 5).

Distinct patterns of BCL-2 family dependency have been observed across malignancies treated with nucleoside analogs. In AML, resistance to Ara-C is commonly associated with increased reliance on anti-apoptotic proteins BCL-2 and MCL-1. Similarly, in solid tumors such as PDAC, metabolic rewiring toward elevated OXPHOS and redox buffering frequently coincides with the upregulation of anti-apoptotic BCL-2 family proteins, reinforcing survival under GEM-induced stress. These adaptations underscore the role of BCL-2 family proteins as convergence points linking mitochondrial metabolism, stress signaling, and cell survival [116].

Together, these findings establish anti-apoptotic BCL-2 family proteins as key survival factors downstream of mitochondrial dysfunction and metabolic stress induced by nucleoside analogs. Therapeutic strategies that disrupt anti-apoptotic buffering have the potential to restore apoptotic sensitivity by lowering the apoptotic threshold, thereby converting persistent replication and metabolic stress into irreversible cell death. As such, targeting BCL-2 family dependencies represents a rational and complementary approach to mitochondria- and DDR-based combination strategies aimed at overcoming nucleoside analog resistance.

### 6.4. Targeting BCL-2 with Venetoclax + Nucleoside Analogs

The selective BCL-2 inhibitor venetoclax (VEN) is the most clinically advanced agent in this class and has rapidly become the backbone of low-intensity regimens for older or unfit AML patients. Preclinical studies in Ara-C-resistant leukemic models demonstrated that BCL-2 upregulation is a hallmark of resistance and that VEN can restore chemosensitivity, showing synergistic cytotoxicity with deoxycytidine analogs and partially reversing resistance in cells overexpressing BCL-2 [117]. These observations provided a mechanistic rationale for combining VEN with nucleoside analogs in the clinic.

Clinically, the combination of VEN with AZA (VEN-AZA) in newly diagnosed, older adults with AML that are ineligible for intensive induction chemotherapy has transformed the landscape of first-line therapy for AML. In the phase III trial, VEN-AZA significantly improved complete remission (CR) rates and overall survival compared with AZA alone, leading to regulatory approval of this regimen and establishing VEN-AZA as a preferred first-line option for this patient population [118,119]. In parallel, a phase III and earlier phase Ib work showed that VEN combined with low-dose Ara-C (LDAC) yields rapid and durable remissions with a manageable safety profile in older AML patients that are not eligible for intensive chemotherapy, supporting VEN + LDAC as another low-intensity first-line therapy for this patient population [120,121].

Building on these successes, several groups have extended VEN combinations to more cytotoxic nucleoside analog-based regimens. VEN has been combined with standard daunorubicin-Ara-C “7+3” induction and with CdA-Ara-C-based intensive regimens, producing high CR/CRi (complete remission with incomplete blood recovery) rates and encouraging survival in both newly diagnosed and relapsed/refractory AML [120,122,123]. More recently, lower-intensity CdA/LDAC/VEN and related quadruplet strategies incorporating AZA have shown promising activity in both newly diagnosed and relapsed/refractory AML cohorts, including patients that were previously exposed to VEN-HMA, further underscoring the value of layering the BCL-2 blockade onto nucleoside analog platforms [124].

Other BCL-2 family members, MCL-1 and BCL-XL, have remained challenging to target, due to off-target effects including cardiac toxicities and thrombopenia, respectively [125]. Therefore, the pharmacologic disruption of BCL-2 dependency with VEN effectively lowers this apoptotic threshold, converting sublethal replication stress into cell death and offering a powerful strategy to overcome, or at least delay, resistance to nucleoside analog-based therapies.

## 7. Conclusions

Together, this summation of the literature highlights that nucleoside analog resistance is not an untreatable endpoint but a pharmacologically actionable state. The multiple layers of resistance to nucleoside analogs, ranging from reduced drug uptake, attenuated drug activation due to loss of dCK, dNTP imbalance, SAMHD1-mediated hydrolysis of the triphosphate metabolite, reprogrammed metabolic pathways promoting cell survival, and upregulation of anti-apoptotic Bcl-2 proteins, can now be targeted through rational drug-delivery platforms and rationally designed combination therapies. These approaches are emerging as powerful tools to prevent, delay, or overcome therapeutic failure.

Looking forward, future clinical directions will likely center several key areas. First, the continued development of selective inhibitors that directly target resistance-driving mechanisms will be essential for translating preclinical findings into improved patient outcomes. Second, integrating these mechanistic insights into patient stratification frameworks, such as screening for SAMHD1 expression, RNR dependence, metabolic signatures, or transporter status, will help to identify which patients are most likely to benefit from specific therapeutic strategies. Third, pairing nucleoside analogs with targeted agents, metabolic modulators, or engineered delivery systems should be tested in early-phase clinical trials to evaluate safety, pharmacodynamics, and their ability to prolong survival. As these therapeutic strategies further develop, they have the potential to shift nucleoside analog therapy toward more individualized, mechanism-informed patient care.

## Figures and Tables

**Table 1:** US FDA-approved nucleoside analogs for cancer treatment, their active metabolites, and main mechanism(s) of action.

Drug	FDA Approval	Cancer Use	Mechanism of Action
Gemcitabine (GEM)	1996	Pancreatic cancer	Inhibition of DNA synthesis Inhibition of ribonucleotide reductase (RNR)
	1998	Non-small cell lung cancer	
	2004	Breast cancer	
Fluorouracil (5-FU)	1962	Adenocarcinoma of the colon, rectum, breast, stomach, and pancreas	Inhibition of RNA processing Inhibition of thymidylate synthase
Cytarabine (Ara-C)	1969	Acute myeloid leukemia Acute lymphocytic leukemia	Inhibition of DNA synthesis
Azacitidine (AZA)	2004	Myelodysplastic syndrome	Inhibition of RNA synthesis
	2020	Acute myeloid leukemia	Inhibition of DNA methyltransferase (DNMT), causing DNA hypomethylation
Decitabine (DAC)	2006	Myelodysplastic syndrome	Inhibition of DNMT causing DNA hypomethylation
Cladribine (CdA)	1993	Hairy cell leukemia	Inhibition of DNA synthesis
Fludarabine (FA)	1991	B-cell chronic lymphocytic leukemia	Inhibition of DNA synthesis Inhibition of DNA polymerase, RNR, and DNA primase
Nelarabine (Ara-G)	2006	T-cell acute lymphoblastic leukemia T-cell lymphoblastic lymphoma	Inhibition of DNA synthesis

**Table 2:** Nanoparticles are synthesized, containing nucleoside analogs + SMI to bypass the transport barrier and increase cytotoxicity.

Nanoparticle	Therapeutic Cargos	Purpose	Key Results
Mesoporous Silica Nanoparticle/5FU- Everolimus@ chitosan hydrogels (MSN/5FU-EVE@CSH)	5-FU and EVE	MSN improves drug targeting and therapeutic efficacy by exhibiting high surface-to-volume ratio 5-FU inhibits DNA synthesis EVE targets mTOR pathway CSH sustains drug release	The combination treatment groups loaded on MSN and CSH nanoparticles showed a significant reduction in tumor size and lung metastasis compared to the monotherapy and control groups [32].
Hyaluronic-acid-modified zeolitic imidazolate framework @benproperine phosphate/GEM (HA/ZIF-8@BPP/Gem)	GEM and BPP	BPP initiated autophagy but blocked autophagosome-lysosome fusion; thereby, GEM-induced protective autophagy was turned into a lethal autophagy.	Represents an efficient drug-repurposing nanoplatform for delivering a promising drug combination, which may sensitize pancreatic cancer to chemotherapy and improve patient survival outcomes [33].
GE11 peptide-modified polyphenol-iron chelate nanoparticles/GEM- leflunomide (GE11-PEG-pPCA/Gem-Lef)	GEM and leflunomide	Leflunomide inhibits dihydroorotate dehydrogenase (DHODH) that is upregulated in GEM resistance	Demonstrated superior antitumor efficacy compared to the standard of care regimen. GEM concentration was lowered 6.3-fold in nanoparticles with increased benefit compared to free drug [34].
Nanostructured lipid carrier- Docetaxel/5FU (NLC-DCT/5-FU)	5-FU and DCT	DCT and 5-FU strongly cooperate to stop cell proliferation and induce cell death	In vitro cytotoxicity assays showed significantly lower IC_50_ values for nanoparticle combination compared to free DOX and FU. In vivo studies showed significantly reduced tumor size and low toxicity [35].
Generation 4 (G4) acetyl-terminated poly(amidoamine) dendrimers conjugated with folic acid: 5FU: Sorafenib (G4ACE-FA:5-FU:SF)	5-FU and SF	G4ACE-FA sustains strong drug binding and targeted release to tumor. SF is a multi-kinase inhibitor that suppresses angiogenesis and tumor proliferation. Combining with FU can synergize to kill cancer cells	Nanoparticles were successfully synthesized using a folic-acid-based platform. Use of this platform demonstrated synergy with the loaded cargo, offering a promising platform to deliver anti-cancer drugs [36].
Navitoclax/decitabine nanoparticles (NAV/DCB NPs)	DAC and NAV	NAV is a pan BCL-2 inhibitor that synergizes with DAC in solid and liquid tumors	In vitro and in vivo evaluation of NAV/DCB demonstrated synergistic effects, increased drug accumulation, and decreased toxicity compared to free drug in both AML and breast cancer models [37].

## Data Availability

No new data were created for this review article.
